# Association Between Alcohol Consumption and the Risk of Type 2 Diabetes Mellitus Across Different Body Mass Index Categories Among Japanese Workers

**DOI:** 10.2188/jea.JE20240259

**Published:** 2025-08-05

**Authors:** Yu Wang, Yosuke Inoue, Shohei Yamamoto, Ami Fukunaga, Shuichiro Yamamoto, Toru Honda, Tohru Nakagawa, Takeshi Hayashi, Maki Konishi, Tetsuya Mizoue

**Affiliations:** 1 Department of Epidemiology and Prevention, Center for Clinical Sciences, Japan Institute for Health Security, Tokyo, Japan; 2 Department of Public Health and Health Policy, Graduate School of Medicine, Hiroshima University, Hiroshima, Japan; 3 Hitachi, Ltd, Ibaraki, Japan

**Keywords:** alcohol consumption, type 2 diabetes mellitus, body mass index

## Abstract

**Background:**

While evidence from Western countries links low-to-moderate alcohol consumption to a lower risk of type 2 diabetes mellitus (T2D), findings have been inconsistent in Asia. Since T2D in Asia involves both insulin resistance and deficient insulin secretion, both of which are differently affected by alcohol, we prospectively examined whether the association differs according to body mass index (BMI) categories among the Japanese.

**Methods:**

Participants were 31,524 health checkup examinees (26,819 males and 4,705 females aged 20–64 years) who were free from diabetes at baseline. Self-reported data on alcohol use were used to estimate the average daily alcohol consumption at the baseline. Incident diabetes was identified at annual checkups during the follow-up period. A Cox proportional hazards model was used to estimate hazard ratios and 95% confidence intervals.

**Results:**

During a median follow-up of 12.0 years, 3,527 male and 287 female participants developed T2D. The association between alcohol consumption and T2D risk differed markedly by BMI in both sexes. Among males, low- and moderate-level alcohol consumption was associated with a lower T2D risk in individuals with BMI ≥25.0 kg/m^2^, whereas consumption at a level of 2 *go*/day (approximately 46 g ethanol) was linked to an increased T2D risk in those with BMI ≤22.0 kg/m^2^. In females, similar patterns were observed, although confidence intervals were broad due to the smaller sample size.

**Conclusion:**

In Japan, low-to-moderate alcohol consumption may lower T2D risk in those with excess body weight, while high alcohol consumption may increase T2D risk in those with lower BMI.

## INTRODUCTION

Previous research exploring the association between alcohol consumption and type 2 diabetes mellitus (T2D) has yielded inconsistent findings across different racial/ethnic groups.^1^^,^^2^ A meta-analysis of 26 studies, the majority of which were conducted in Western countries, reported a U-shaped association between alcohol consumption and T2D, with the lowest risk of T2D observed among light and moderate alcohol drinkers.^1^ In contrast, a recent meta-analysis of prospective studies among Asian men suggested that light and moderate alcohol consumption does not necessarily reduce T2D risk, whereas heavy alcohol consumption is associated with an increased T2D risk.^2^

A possible explanation for the discrepancy in the association between alcohol consumption and T2D risk between Western and Asian studies may originate from the difference in the average body size between these populations, coupled with the varying effects of alcohol consumption on insulin resistance and insulin secretion, two fundamental pathophysiological features in the development of T2D.^1^^,^^3^ Light-to-moderate alcohol consumption can decrease insulin resistance,^4^ and this effect may be more evident among Western populations, who have a higher prevalence of obesity (and insulin resistance) than Asian populations.^5^ In contrast, insulin secretion, which has been suggested to be impaired by alcohol consumption,^6^ is notably lower in Asians than in Westerners. This reduced buffering ability against alcohol intake among Asians may increase their risk of developing T2D, even at low levels of alcohol consumption.

This study was designed to extend the findings of previous studies for various reasons. First, it is unclear whether the association between alcohol consumption and T2D risk varies according to body mass index (BMI) categories within a population. The existing limited studies have been summarized, for example, in a systematic review of four Japanese studies,^7^ two of which revealed a positive association between alcohol consumption and T2D in individuals with a lower BMI, along with a U-shaped^8^ or inverse association^9^ in individuals with a higher BMI. In contrast, one study indicated a U-shaped association across both lower and higher BMI categories,^10^ whereas the remaining study identified a positive association in lean individuals and a non-significant positive association in individuals with a higher BMI.^11^ Second, the previous studies mentioned above gathered the baseline information in the 1980s–90s, despite the significant increase in overweight or obesity prevalence among Asian populations, especially in Japan, over the past few decades.^12^ Moreover, the pattern of alcohol consumption has recently changed.^13^^,^^14^ In light of these changes, it has become imperative to reevaluate the association between alcohol consumption and the risk of T2D in Japan using an updated dataset. In fact, a recent study from Japan,^15^ with baseline data collected in 2020–2021, reported an association similar to patterns observed in Western countries.

Thus, the present study aimed to examine whether the association between alcohol consumption and T2D risk differs according to BMI categories among Japanese participants, using baseline information collected in 2008. We hypothesized that increased alcohol consumption is associated with an increased T2D risk among individuals with a lower BMI, whereas moderate alcohol consumption is linked to a decreased T2D risk among individuals with a higher BMI.

## METHODS

### Study design

This study was part of the Japan Epidemiology Collaboration on Occupational Health (J-ECOH) Study, an ongoing multicompany study of workers in Japan. Data used for the present study were derived from one participating company specializing in the manufacture of electrical machinery and apparatus. We chose this company because it provided detailed questionnaire information on alcohol consumption. We also had access to information collected during annual health checkups and questionnaire data on various covariates, consistent with other J-ECOH study companies. We used the 2008 health checkup data (between April 2008 and March 2009) as the baseline. The details of the related information have been described elsewhere.^16^^,^^17^

Ethical approval for the study protocol was obtained from the ethics committee of the Japan Institute for Health Security (NCGM-S-001140). Participants were enrolled unless they chose to opt out, as communicated through the internal bulletin boards of the company. Instead of obtaining individual informed consent, we informed the workers about the study’s aims and procedures, with the option to withdraw participation at any point. This procedure adhered to the Act on the Protection of Personal Information and Ethical Guidelines for Medical and Biological Research Involving Human Subjects.

### Study participants

**Figure 1.**
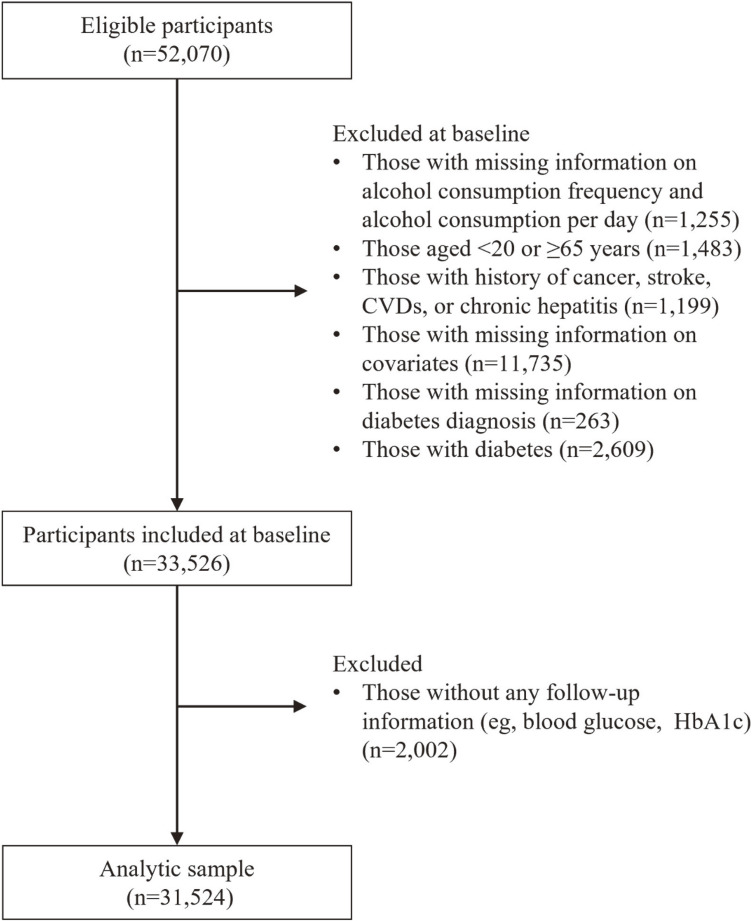
Selection of participants. BMI, body mass index; CVD, cardiovascular disease; HbA1c, glycated hemoglobin.

As shown in Figure 1, 52,070 individuals participated in the 2008 health checkup. Of these, we excluded (1) those with missing information on alcohol consumption frequency and amount per occasion (*n* = 1,255); (2) those aged <20 years or ≥65 years (*n* = 1,483); (3) those who self-reported to have a history of cancer, stroke, cardiovascular diseases, or chronic hepatitis (*n* = 1,199); (4) those with missing information on covariates (*n* = 11,735; BMI [*n* = 1]; job grade [*n* = 3,473]; marital status [*n* = 540]; smoking [*n* = 53]; occupational physical activity [*n* = 262]; leisure-time physical activity [*n* = 975], and dyslipidemia [*n* = 6,431]); (5) those with missing information at least one of the following three information to define diabetes diagnosis at baseline (ie, plasma glucose, glycated hemoglobin [HbA1c], and antidiabetic drug therapy; *n* = 263); and (6) those with diabetes at baseline (determined using fasting plasma glucose ≥126 mg/dL, random plasma glucose ≥200 mg/dL, HbA1c ≥6.5%, or antidiabetic drug therapy; *n* = 2,609). Plasma glucose concentrations were assayed using the glucose electrode technique, and HbA1c levels were measured using high-performance liquid chromatography. For data collected on or before March 31, 2013, HbA1c values were converted from Japan Diabetes Society (JDS) units to National Glycohemoglobin Standardization Program (NGSP) units using the equation: NGSP (%) = 1.02 × JDS (%) + 0.25. We then excluded those who did not participate in any of the subsequent annual health checkups (*n* = 2,002), leaving 31,524 participants for the analyses.

### Ascertainment of incident T2D and follow-up of participants

Incident T2D was defined when individuals were first diagnosed with diabetes at annual health checkups based on the American Diabetes Association criteria: fasting plasma glucose ≥126 mg/dL, random plasma glucose ≥200 mg/dL, or HbA1c ≥6.5%.^18^ Of the 287,383 observations included in the analysis, fasting blood data were available for 278,350 (96.9%) observations, and HbA1c data were available for 280,968 (97.8%) observations. In addition, those who self-reported to undergo antidiabetic treatment were also considered to have T2D.

We followed the participants for incident T2D until March 31, 2022. For those who developed T2D, person-years were calculated from the date of the baseline health examination until the date of a subsequent health examination in which they were first judged to be diabetic. Participants who did not develop T2D during the study period were censored as of their last health examination, with March 31, 2022, as the potential final follow-up date.

### Assessment of average daily alcohol consumption

Questionnaire information on alcohol consumption frequency (measured in the number of days per week) and alcohol consumption per day (measured in ‘*go*,’ a traditional Japanese unit of volume equivalent to approximately 23.0 g of ethanol) was used to assess the average daily alcohol consumption. Specifically, we calculated this by multiplying alcohol consumption frequency by alcohol consumption per day and then dividing the product by 7. Using cut-offs of 0, 0.5, 1, and 2 *go*/day, participants were then categorized into the following groups based on their alcohol consumption: 0, 0.1–0.5, 0.6–1.0, 1.1–2.0, and >2.0 *go*/day. The corresponding ranges for alcohol consumption categories, from lowest to highest, were 0; 0.1–11.5; 11.6–23.0; 23.1–46.0; and >46.0 g ethanol/day.

### Assessment of BMI

Body height was measured with participants in a standing position, and body weight was measured using a scale, with participants wearing light clothing and no shoes. BMI was calculated as weight (kg) divided by height squared (m^2^). We categorized participants into the following BMI categories: ≤22.0, 22.1–24.9, ≥25.0 kg/m^2^.

### Covariates

The following sociodemographic and lifestyle-related covariates were assessed using questionnaire information collected at baseline: marital status (married or unmarried [single, divorced, or widowed]), job position (high or low), smoking status (never, former, or current), occupational physical activity, leisure-time physical activity, and family history of diabetes. Occupational physical activity was assessed by asking participants to select the option that best described their typical activity level at work: mostly sitting, mostly standing, mostly walking, or mostly physically active. Leisure-time physical activity was measured by asking participants about the frequency and duration of up to three activities from a list of 20 (eg, baseball, golf, walking, and tennis). The total time spent per week was calculated and categorized into 0, 1–59, 60–119, or ≥120 minutes per week. A family history of diabetes was defined as having either a parent or sibling diagnosed with the condition. Participants were asked to select from a list of seven diseases (including diabetes) that their parents or siblings had been diagnosed with. Those who selected diabetes were categorized as having a family history of diabetes (yes or no).

Hypertension was defined as systolic blood pressure ≥140 mm Hg, diastolic blood pressure ≥90 mm Hg, or receiving medical treatment for hypertension. Dyslipidemia was defined as triglyceride level ≥150 mg/dL, low-density lipoprotein cholesterol level ≥140 mg/dL, high-density lipoprotein cholesterol level <40 mg/dL, or receiving medical treatment for dyslipidemia.

### Statistical analysis

We examined the association between alcohol consumption and the risk of T2D using a Cox proportional hazards regression model. In model 1, we adjusted for age (years, continuous). We further adjusted for marital status, job position, smoking status, occupational physical activity, leisure-time physical activity, family history of diabetes, hypertension, and dyslipidemia in model 2 and BMI (continuous) in model 3. We calculated the *P*-values for linear and quadratic trends using the coefficients of orthogonal polynomials. We confirmed that there was no violation of the proportional hazards assumption, as indicated with Schoenfeld residual tests.

In stratified analyses according to BMI categories (≤22.0, 22.1–24.9, and ≥25.0 kg/m^2^), we adjusted for the same variables as in the first two models. We also tested the potential interactions between alcohol consumption and BMI categories by assessing the likelihood ratio tests in models with and without the cross-product terms (BMI categories × alcohol consumption categories).

To illustrate the dose-response relationship between alcohol consumption and the risk of T2D among males, we drew restricted cubic spline curves for all participant groups as well as for participants with BMI ≤22.0 kg/m^2^, those with BMI 22.1–24.9 kg/m^2^, and those with BMI ≥25.0 kg/m^2^. These curves were plotted with three knots placed at the 5th, 50th, and 95th percentiles within the range of 0 to 3 *go*/day (equivalent to 99th percentile value). The linear trend was assessed by examining the *P*-value obtained when alcohol consumption was modeled as a continuous variable while the non-linear trend was evaluated using the *P*-value from the likelihood ratio test, which compared models with and without the inclusion of spline terms.

All statistical analyses were performed using Stata version 17.0 (StataCorp, College Station, TX, USA). The results are presented as hazard ratios (HRs) with corresponding 95% confidence intervals (CIs).

## RESULTS

Table 1 and Table 2 present the basic characteristics of the male and female study participants, respectively, categorized according to alcohol consumption, while eTable 1 provides the baseline characteristics of the combined male and female participants. The mean age was 43.8 (standard deviation [SD], 9.3) years, with participants who consumed more alcohol being older than those who consumed less alcohol. The proportion of male participants was 85.1%, and this was higher among those who consumed more alcohol than among those who consumed less alcohol. Furthermore, participants who consumed more alcohol tended to hold high job positions, were current smokers, and had hypertension. Family history of diabetes, BMI, and dyslipidemia did not differ significantly across alcohol consumption categories.

**Table 1.** Baseline characteristics of male participants according to average daily alcohol consumption category

Characteristics	Male participants	Alcohol consumption categories (*go*/day)^a^				

		0	0.1–0.5	0.6–1.0	1.1–2.0	>2.0
*N*	26,819	7,476	7,162	5,782	5,296	1,103
Age, years, mean [SD]	43.9 [9.2]	42.3 [9.6]	42.2 [9.1]	45.0 [8.8]	46.8 [8.4]	46.7 [8.1]
Married, *n* (%)	20,625 (76.9)	5,118 (68.5)	5,461 (76.3)	4,785 (82.8)	4,417 (83.4)	844 (76.5)
High job position, *n* (%)	6,119 (22.8)	1,046 (14.0)	1,716 (24.0)	1,563 (27.0)	1,482 (28.0)	312 (28.3)
Smoking status, *n* (%)						
Never	9,231 (34.4)	3,017 (40.4)	3,008 (42.0)	1,808 (31.3)	1,196 (22.6)	202 (18.3)
Former	5,440 (20.3)	1,080 (14.5)	1,395 (19.5)	1,356 (23.5)	1,350 (25.5)	259 (23.5)
Current	12,148 (45.3)	3,379 (45.2)	2,759 (38.5)	2,618 (45.3)	2,750 (51.9)	642 (58.2)
Occupational physical activity, *n* (%)						
Mostly sedentary	16,430 (61.3)	4,074 (54.5)	4,821 (67.3)	3,700 (63.4)	3,183 (60.1)	652 (59.1)
Mostly standing	3,431 (12.8)	1,200 (16.1)	745 (10.4)	673 (11.6)	660 (12.5)	153 (13.9)
Mostly walking	4,874 (18.2)	1,460 (19.5)	1,133 (15.8)	1,045 (18.1)	1,036 (19.6)	200 (18.1)
Mostly physically active	2,084 (7.8)	742 (9.9)	463 (6.5)	364 (6.3)	417 (7.9)	98 (8.9)
Leisure-time physical activity, min/week, *n* (%)						
0	16,845 (62.8)	5,079 (67.9)	4,349 (60.7)	3,524 (61.0)	3,168 (59.8)	725 (65.7)
1–59	2,455 (9.2)	622 (8.3)	790 (11.0)	525 (9.1)	445 (8.4)	73 (6.6)
60–119	2,653 (9.9)	595 (8.0)	803 (11.2)	619 (10.7)	541 (10.2)	95 (8.6)
≥120	4,866 (18.1)	1,180 (15.8)	1,220 (17.0)	1,114 (19.3)	1,142 (21.6)	210 (19.0)
Family history of diabetes, *n* (%)	3,794 (14.2)	1,054 (14.1)	997 (13.9)	817 (14.1)	751 (14.2)	175 (15.9)
Body mass index, kg/m^2^, mean [SD]	23.5 [3.2]	23.6 [3.5]	23.5 [3.2]	23.4 [2.9]	23.4 [2.8]	23.4 [3.0]
Hypertension, *n* (%)	4,137 (15.4)	875 (11.7)	844 (11.8)	920 (15.9)	1,227 (23.2)	271 (24.6)
Dyslipidemia, *n* (%)	12,647 (47.2)	3,885 (52.0)	3,322 (46.4)	2,545 (44.0)	2,397 (45.3)	498 (45.2)
Fasting plasma glucose, mean [SD]^b^	97.2 [9.0]	95.6 [8.8]	96.2 [8.8]	97.7 [8.7]	99.4 [9.2]	99.2 [9.3]
HbA1c, mean [SD]	5.57 [0.32]	5.60 [0.32]	5.57 [0.32]	5.57 [0.31]	5.57 [0.32]	5.52 [0.33]

SD, standard deviation.

^a^One *go* of Japanese sake contains approximately 23 g of ethanol. The corresponding ranges for alcohol consumption categories, from lowest to highest, were 0; 0.1–11.5 g; 11.6–23.0 g; 23.1–46.0 g; and ≥46.1 g.

^b^Information was available for 25,332 participants, consisting of 6,863, 6,717, 5,571, 5,121, and 1,060 participants who consumed 0, 0.1–0.5, 0.6–1.0, 1.1–2.0, and >2.0 *go*/day of alcohol, respectively.

**Table 2.** Baseline characteristics of female participants according to average daily alcohol consumption category

Characteristics	Female participants	Alcohol consumption categories (*go*/day)^a^			

		0	0.1–0.5	0.6–1.0	>1.0
*N*	4,705	3,055	1,178	324	148
Age, years, mean [SD]	43.2 [9.4]	44.1 [9.5]	40.8 [9.1]	42.9 [8.4]	43.3 [8.1]
Married, *n* (%)	2,835 (60.3)	1,886 (61.7)	686 (58.2)	192 (59.3)	71 (48.0)
High job position, *n* (%)	42 (0.9)	23 (0.8)	12 (1.0)	4 (1.2)	3 (2.0)
Smoking status, *n* (%)					
Never	3,834 (81.5)	2,634 (86.2)	928 (78.8)	209 (64.5)	63 (42.6)
Former	238 (5.1)	105 (3.4)	84 (7.1)	31 (9.6)	18 (12.2)
Current	633 (13.5)	316 (10.3)	166 (14.1)	84 (25.9)	67 (45.3)
Occupational physical activity, *n* (%)					
Mostly sedentary	2,389 (50.8)	1,503 (49.2)	657 (55.8)	159 (49.1)	70 (47.3)
Mostly standing	1,147 (24.4)	786 (25.7)	249 (21.1)	77 (23.8)	35 (23.7)
Mostly walking	751 (16.0)	488 (16.0)	182 (15.5)	56 (17.3)	25 (16.9)
Mostly physically active	418 (8.9)	278 (9.1)	90 (7.6)	32 (9.9)	18 (12.2)
Leisure-time physical activity, min/week, *n* (%)					
0	3,462 (73.6)	2,332 (76.3)	803 (68.2)	221 (68.2)	106 (71.6)
1–59	374 (8.0)	209 (6.8)	116 (9.9)	33 (10.2)	16 (10.8)
60–119	370 (7.9)	221 (7.2)	116 (9.9)	23 (7.1)	10 (6.8)
≥120	499 (10.6)	293 (9.6)	143 (12.1)	47 (14.5)	16 (10.8)
Family history of diabetes, *n* (%)	758 (16.1)	478 (15.7)	196 (16.6)	59 (18.2)	25 (16.9)
Body mass index, kg/m^2^, mean [SD]	21.9 [3.5]	22.0 [3.6]	21.8 [3.5]	21.4 [3.2]	21.8 [3.7]
Hypertension, *n* (%)	425 (9.0)	288 (9.4)	88 (7.5)	30 (9.3)	19 (12.8)
Dyslipidemia, *n* (%)	1,144 (24.3)	815 (26.7)	240 (20.4)	56 (17.3)	33 (22.3)
Fasting plasma glucose, mean [SD]^b^	91.3 [8.2]	91.1 [8.2]	91.2 [8.0]	91.4 [8.1]	94.2 [9.5]
HbA1c, mean [SD]	5.54 [0.32]	5.57 [0.32]	5.48 [0.33]	5.45 [0.31]	5.41 [0.33]

SD, standard deviation.

^a^One *go* of Japanese sake contains approximately 23 g of ethanol. The corresponding ranges for alcohol consumption categories, from lowest to highest, were 0; 0.1–11.5 g; 11.6–23.0 g; and ≥23.1 g.

^b^Information was available for 4,246 participants, consisting of 2,769, 1,048, 296, and 133 participants who consumed 0, 0.1–0.5, 0.6–1.0, and >1.0 *go*/day of alcohol, respectively.

During a median follow-up period of 12.0 (range, 0.2–14.0) years, 3,527 male and 287 female participants developed T2D. Among males, the incidence of T2D was 13.8 cases per 1,000 person-years, with rates of 14.1, 12.2, 12.6, 16.3, and 17.7 across increasing alcohol consumption categories. For females, the overall incidence was 6.7 cases per 1,000 person-years, with rates of 7.3, 5.6, 12.2, and 10.3 from the lowest to highest alcohol consumption categories. Participants with a BMI ≥25 kg/m^2^ generally had a higher T2D incidence than those with a lower BMI in both sexes.

In Table 3, the Cox proportional hazards model, which examined the association between alcohol consumption categories and the risk of T2D in male participants, showed that the HRs for those consuming 0.1–0.5 *go*/day, 0.6–1.0 *go*/day, 1.1–2.0 *go*/day, and >2.0 *go*/day were 0.86 (95% CI, 0.78–0.94), 0.79 (95% CI, 0.72–0.88), 0.94 (95% CI, 0.86–1.04), and 1.05 (95% CI, 0.89–1.23), respectively (model 1). The reduced T2D risk associated with alcohol consumption of 0.1–0.5 and 0.6–1.0 *go*/day was attenuated but remained statistically significant among those consuming 0.6–1.0 *go*/day (HR 0.88; 95% CI, 0.79–0.97) after further adjustment for covariates (model 2). This association was further attenuated and became statistically non-significant after additional adjustment for BMI (model 3).

**Table 3.** Hazard ratios and 95% confidence intervals for type 2 diabetes mellitus according to baseline average daily alcohol consumption category among male workers of a Japanese manufacturing company (2008–2022)

	Alcohol consumption categories (*go*/day)^a^					*P*_linear_	*P*_quadratic_

	0	0.1–0.5	0.6–1.0	1.1–2.0	>2.0		
**Whole participants** (*n* = 26,819)							
Person-years	71,706	70,850	54,908	48,282	9,944		
Cases (incidence)^b^	1,011 (14.1)	861 (12.2)	694 (12.6)	785 (16.3)	176 (17.7)		
Model 1	1.00 (ref.)	0.86 (0.78–0.94)	0.79 (0.72–0.88)	0.94 (0.86–1.04)	1.05 (0.89–1.23)	0.27	<0.001
Model 2	1.00 (ref.)	0.94 (0.85–1.03)	0.88 (0.79–0.97)	0.97 (0.88–1.07)	1.00 (0.85–1.18)	0.81	0.050
Model 3	1.00 (ref.)	0.93 (0.85–1.02)	0.91 (0.82–1.00)	1.02 (0.92–1.12)	1.06 (0.90–1.24)	0.27	0.057

**Stratified analysis by BMI categories** ^c^							
**BMI ≤22.0 kg/m^2^** (*n* = 8,959)							
Person-years	25,925	24,622	18,821	15,991	3,458		
Cases (incidence)^b^	144 (5.6)	131 (5.3)	103 (5.5)	158 (9.9)	42 (12.1)		
Model 1	1.00 (ref.)	0.95 (0.75–1.20)	0.83 (0.64–1.07)	1.30 (1.03–1.63)	1.58 (1.12–2.23)	0.001	0.009
Model 2	1.00 (ref.)	1.11 (0.87–1.41)	0.88 (0.68–1.14)	1.26 (1.00–1.59)	1.41 (1.00–1.99)	0.034	0.16
**BMI 22.1**–**24.9 kg/m^2^** (*n* = 10,486)							
Person-years	25,727	27,143	23,123	20,636	3,896		
Cases (incidence)^b^	331 (11.2)	280 (9.7)	268 (11.5)	320 (15.2)	58 (14.9)		
Model 1	1.00 (ref.)	0.88 (0.75–1.04)	0.90 (0.76–1.06)	1.10 (0.93–1.29)	1.12 (0.85–1.49)	0.14	0.14
Model 2	1.00 (ref.)	0.95 (0.80–1.13)	0.99 (0.83–1.17)	1.14 (0.96–1.34)	1.08 (0.81–1.44)	0.28	0.75
**BMI ≥25.0 kg/m^2^** (*n* = 7,374)							
Person-years	20,054	19,085	12,964	11,655	2,590		
Cases (incidence)^b^	580 (28.9)	466 (24.4)	326 (25.1)	313 (26.9)	76 (29.3)		
Model 1	1.00 (ref.)	0.83 (0.74–0.94)	0.82 (0.71–0.94)	0.84 (0.73–0.96)	0.93 (0.74–1.18)	0.57	0.029
Model 2	1.00 (ref.)	0.89 (0.79–1.01)	0.88 (0.76–1.01)	0.87 (0.75–1.00)	0.94 (0.74–1.20)	0.55	0.16

BMI, body mass index.

Model 1: Adjusted for age (years, continuous).

Model 2: Model 1 + marital status (married or unmarried), job position (high or low), smoking status (never, former, or current), occupational physical activity (mostly sedentary, mostly standing, mostly walking, or mostly physically active), leisure-time physical activity (0, 1–59, 60–119, or ≥120 min/week), family history of diabetes (yes or no), hypertension (yes or no), and dyslipidemia (yes or no).

Model 3: Model 2 + BMI (kg/m^2^, continuous).

^a^One *go* of Japanese sake contains approximately 23 g of ethanol. The corresponding ranges for alcohol consumption categories, from lowest to highest, were 0; 0.1–11.5 g; 11.6–23.0 g; 23.1–46.0 g; and ≥46.1 g.

^b^Incidence is shown in terms of the number of cases per 1,000 person-years of observation.

^c^*P* for interaction is <0.001.

Among male participants with a BMI of ≤22.0 kg/m^2^, those who consumed >2.0 *go*/day of alcohol had a significantly higher risk of T2D than non-drinkers (HR 1.58; 95% CI, 1.12–2.23) in model 1. After adjusting for other variables in model 2, this association was attenuated (HR 1.41; 95% CI, 1.00–1.99). Among participants with a BMI of ≥25.0 kg/m^2^, those who consumed 1.1–2.0 *go*/day of alcohol had a significantly lower risk of T2D than non-drinkers (HR 0.84; 95% CI, 0.73–0.96) in model 1. In model 2, this association was attenuated (HR 0.87; 95% CI, 0.75–1.00). No significant associations were observed among participants with a BMI of 22.1–24.9 kg/m^2^. We observed a significant interaction between alcohol consumption and BMI categories in relation to T2D (*P* for interaction <0.001).

**Figure 2.**
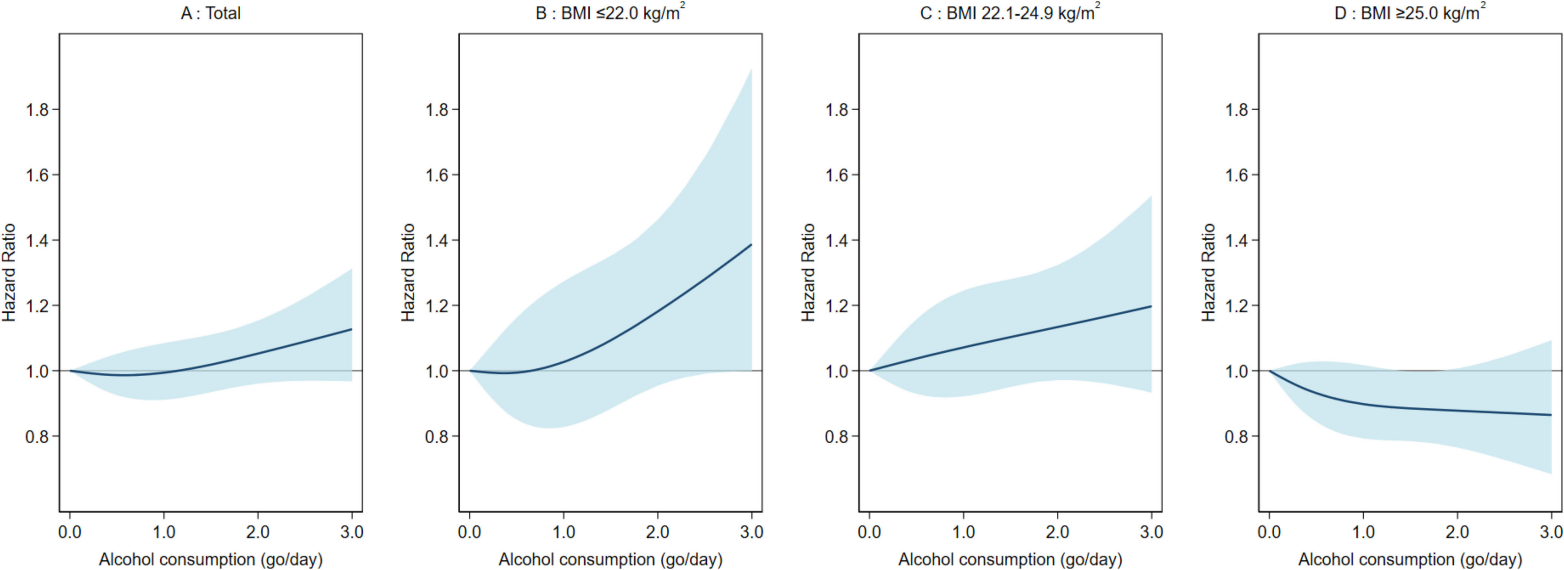
Dose-response association between alcohol consumption and diabetes among male participants. One go of Japanese sake contains approximately 23 g of ethanol. (**A**) among total participants (linear trend, *P* = 0.22; non-linear trend, *P* = 0.36); (**B**) among participants with BMI ≤22.0 kg/m^2^ (*P* = 0.07; *P* = 0.48); (**C**) among participants with BMI = 22.1–24.9 kg/m^2^ (*P* = 0.11; *P* = 0.92); (**D**) among participants with BMI ≥25 kg/m^2^ (*P* = 0.07; *P* = 0.41). The reference value is 0 *go*/day (non-drinkers). The model is adjusted for age (years, continuous), marital status (married or unmarried), job position (high or low), smoking status (never, former, or current), occupational physical activity (mostly sedentary, mostly standing, mostly walking, or mostly physically active), leisure-time physical activity (0, 1–59, 60–119, or ≥120 min/week), family history of diabetes (yes or no), hypertension (yes or no), and dyslipidemia (yes or no). BMI, body mass index.

These associations among male participants were illustrated using cubic spline curves (Figure 2). A major difference was observed between BMI categories below and above 25.0 kg/m^2^. For participants with a BMI ≤22.0 kg/m^2^ and 22.1–24.9 kg/m^2^, the curves generally showed an upward trend, indicating increased risk with higher alcohol consumption, with some minor exceptions. In contrast, for participants with a BMI ≥25.0 kg/m^2^, the curve trended downward, indicating a lower risk of T2D; the risk reduction plateaued at around 1 *go*/day of alcohol and remained stable up to 3 *go*/day, the upper limit of alcohol consumption used in the cubic spline analysis.

Table 4 presents results from the Cox proportional hazards model examining the association between alcohol consumption and T2D risk among female participants. When considering all female participants, HRs for alcohol consumption categories, from lowest to highest, were 0.89 (95% CI, 0.67–1.19), 0.68 (95% CI, 0.39–1.19), and 1.43 (95% CI, 0.84–2.47) in model 1, with further adjustments also showing non-significant associations.

**Table 4.** Hazard ratios and 95% confidence intervals for type 2 diabetes mellitus according to baseline average daily alcohol consumption category among female workers of a Japanese manufacturing company (2008–2022)

	Alcohol consumption categories (*go*/day)^a^				*P*_linear_	*P*_quadratic_

	0	0.1–0.5	0.6–1.0	>1.0		
**Female participants** (*n* = 4,705)						
Person-years	27,147	11,137	2,891	1,356		
Cases (incidence)^b^	198 (7.3)	62 (5.6)	13 (12.2)	14 (10.3)		
Model 1	1.00 (ref.)	0.89 (0.67–1.19)	0.68 (0.39–1.19)	1.43 (0.84–2.47)	0.36	0.036
Model 2	1.00 (ref.)	0.87 (0.65–1.16)	0.66 (0.37–1.16)	1.15 (0.65–2.02)	0.89	0.096
Model 3	1.00 (ref.)	0.89 (0.66–1.18)	0.69 (0.39–1.21)	1.13 (0.64–1.99)	0.91	0.14

**Stratified analysis by BMI categories** ^c^						
**BMI ≤22.0 kg/m^2^** (*n* = 2,797)						
Person-years	16,512	7,018	1,928	850		
Cases (incidence)^b^	57 (3.5)	14 (2.0)	106 (1.6)	7 (8.2)		
Model 1	1.00 (ref.)	0.76 (0.42–1.37)	0.55 (0.17–1.76)	2.75 (1.25–6.05)	0.046	0.012
Model 2	1.00 (ref.)	0.74 (0.41–1.34)	0.52 (0.16–1.70)	2.46 (1.03–5.84)	0.12	0.015
**BMI 22.1**–**24.9 kg/m^2^** (*n* = 1,133)						
Person-years	6,337	2,623	560	355		
Cases (incidence)^b^	44 (6.9)	16 (6.1)	3 (5.0)	6 (16.9)		
Model 1	1.00 (ref.)	0.99 (0.56–1.78)	0.77 (0.24–2.47)	2.29 (0.97–5.39)	0.12	0.15
Model 2	1.00 (ref.)	1.07 (0.59–1.94)	0.71 (0.21–2.32)	2.07 (0.83–5.16)	0.26	0.20
**BMI ≥25.0 kg/m^2^** (*n* = 775)						
Person-years	4,297	1,495	363	151		
Cases (incidence)^b^	97 (22.6)	32 (21.4)	7 (19.3)	1 (6.6)		
Model 1	1.00 (ref.)	0.98 (0.66–1.47)	0.88 (0.41–1.91)	0.29 (0.04–2.06)	0.21	0.31
Model 2	1.00 (ref.)	0.92 (0.62–1.39)	0.81 (0.37–1.78)	0.15 (0.02–1.10)	0.058	0.14

BMI, body mass index.

Model 1: Adjusted for age (years, continuous).

Model 2: Model 1 + marital status (married or unmarried), job position (high or low), smoking status (never, former, or current), occupational physical activity (mostly sedentary, mostly standing, mostly walking, or mostly physically active), leisure-time physical activity (0, 1–59, 60–119, or ≥120 min/week), family history of diabetes (yes or no), hypertension (yes or no), and dyslipidemia (yes or no).

Model 3: Model 2 + BMI (kg/m^2^, continuous).

^a^One *go* of Japanese sake contains approximately 23 g of ethanol. The corresponding ranges for alcohol consumption categories, from lowest to highest, were 0; 0.1–11.5 g; 11.6–23.0 g; and ≥23.1 g.

^b^Incidence is shown in terms of the number of cases per 1,000 person-years of observation.

^c^*P* for intetection is 0.022.

When the analysis was stratified by BMI category, alcohol consumption >1.0 g was associated with a higher T2D risk among individuals with a BMI <22.0 kg/m^2^ (HR 2.75; 95% CI, 1.25–6.05), remaining significant after adjustment for covariates in model 2. Although the other associations did not reach statistical significance and confidence intervals were broad, their trends were consistent with those observed in male participants.

## DISCUSSION

In this working population in Japan, male participants consuming 0.6–1.0 *go*/day, equivalent to approximately 11.6–23.0 g of ethanol/day, had a lower risk of T2D compared to non-drinkers. Stratified analysis according to BMI categories revealed that this decreased risk with low- and moderate-level alcohol consumption was more pronounced in individuals with a BMI of ≥25.0 kg/m^2^. Conversely, among participants with a BMI of ≤22.0 kg/m^2^, those consuming >2.0 *go*/day of alcohol tended to exhibit an increased risk of T2D compared to non-drinkers. Among female participants, although associations were non-significant due to wide confidence interval and a small sample size, the associations trended in the same direction as observed among male participants. These findings indicate that the association between alcohol consumption and T2D risk differs depending on the BMI of population subgroups, even within a population.

The increased T2D risk associated with high alcohol consumption among lean individuals in both males and females in our study aligned with that observed in previous research in Japanese cohorts^8^^,^^9^^,^^11^ and a Korean study.^19^ These studies similarly indicated a heightened T2D risk in lean individuals consuming alcohol. For example, Baik et al^19^ analyzed 2,366 lean Korean individuals (defined as BMI <23.0 kg/m^2^) who were aged 40–69 years, reporting HRs for T2D of 1.74, 2.09, and 1.94 for consuming 16–30 g/day, 31–60 g/day, and >60 g/day, respectively, compared with lifetime non-drinkers. Individuals with a lower BMI inherently have a lower insulin secretion capacity,^20^ which may be further impaired by the adverse effects of alcohol on insulin secretion.^21^^–^^23^ Moreover, these individuals are unlikely to experience the advantageous effects of alcohol on insulin resistance, given their general absence of insulin resistance.

Our finding that low-to-moderate alcohol consumption was associated with a lower risk of T2D among individuals with a BMI of ≥25.0 kg/m^2^, with risk reduction plateauing at around 1 *go*/day of alcohol and remained stable up to at least 3 *go*/day, aligned with the results of the meta-analysis of 26 studies,^1^ predominantly based in Western countries. While excessive alcohol consumption can impair insulin secretion in individuals with a higher BMI, similar to that observed in individuals with a lower BMI, individuals with a higher BMI may also experience a protective effect of low to moderate alcohol consumption on insulin resistance because of their typically high baseline insulin resistance.^24^ The varying effects of alcohol consumption on both insulin resistance and insulin secretion may have contributed to the observed association between alcohol consumption and the risk of T2D in individuals with a higher BMI. It should be noted that the HR in Table 3 for the highest consumption category, which approached 1, may have been attributed to some participants consuming more than 3 *go*/day of alcohol. Among females, a reduction in T2D risk with higher alcohol consumption was similarly observed in groups with higher BMI; however, further analysis with a larger sample size would be needed to confirm this finding.

Although a lower risk of T2D associated with low-to-moderate alcohol consumption was not strongly observed among overall participants in this study, the presence of such an association in individuals with a higher BMI, paralleling the findings primarily from Western countries, suggests that the association between alcohol consumption and the risk of T2D in Japan may converge toward the Western pattern if obesity rates continue to increase. Notably, a recent Japanese study involving 10,631 male participants (average age, 47.9 years and average BMI, 23.4 kg/m^2^), which did not stratify their analysis according to BMI categories, also indicated a significantly lower T2D risk with moderate alcohol consumption.^15^

Positioning this study within the context of previous Japanese research, we should also address the discrepancy with the findings of Waki et al,^11^ which showed a non-significant but positive association among the high BMI group. One possible explanation is that the high BMI group in Waki et al’s study may have included fewer individuals with severe obesity compared to our study. This could account for the lack of a reduced risk of T2D associated with low-to-moderate alcohol consumption observed in their findings. If the high BMI group had included more individuals with obesity or severe obesity, a reduction in T2D risk with low-to-moderate alcohol consumption might have been observed, as seen in our study and the other Japanese research.^8^^–^^10^ However, since BMI values within each BMI group were not reported in these previous studies, this hypothesis remains speculative and requires further investigation.

It is critical to note that the observed lower risk of T2D associated with alcohol consumption in individuals with a higher BMI does not imply a recommendation for moderate alcohol consumption in individuals with a higher BMI; moreover, this finding does not suggest obesity combined with alcohol intake as a preventive strategy for T2D. Our data, which illustrate incidence rates across various BMI and alcohol consumption categories, indicate higher incidence rates in those with a BMI of ≥25 kg/m^2^ than in those with a BMI of ≤22 kg/m^2^. Specifically, incidence rates in the BMI ≥25 kg/m^2^ group were 26.5 and 21.7 per 1,000 person-years for male and female participants, respectively, whereas in the BMI ≤22 kg/m^2^ group, the corresponding figures were 6.5 and 3.1 per 1,000 person-years. Thus, our findings must be interpreted with the understanding that obesity is a significant independent risk factor for diabetes. Supporting this, a recent Mendelian randomization analysis of 408,540 individuals of European ancestry from the UK Biobank suggested that consuming over 14 drinks per week (equivalent to >28 g/day of alcohol) can lead to an increase in BMI and waist-to-hip ratio and potentially elevate the risk of diabetes.^25^

### Strengths and limitations

The major strengths of the present study included a longitudinal study design with a maximum of 14 years of follow-up, ascertainment of T2D using annual measurements of plasma glucose and HbA1c levels collected through routine health check-ups, and comprehensive adjustment for covariates. However, this study had several limitations. First, as the data on alcohol consumption quantity and frequency were self-reported exclusively at baseline, there is a possibility of underreporting, particularly among heavy drinkers, which may have introduced misclassification bias. Furthermore, the time-varying nature of alcohol consumption was not accounted for in this study. The degree to which individuals reduce their alcohol consumption following the diagnosis of a condition potentially linked to T2D may influence the U-shaped association observed in this study. Specifically, if a greater number of individuals reduce their alcohol intake to low-to-moderate levels, the U-shaped curve may become shallower; conversely, if complete cessation of alcohol consumption is more prevalent, the curve may deepen. Second, and related to the point above, the inability to separate ex-drinkers from lifetime abstainers in our study may have led to an overestimation of diabetes risk in the non-drinkers group, increasing the apparent magnitude of the association among low to moderate drinkers. Third, our study did not examine the specific types of alcoholic beverages consumed. Previous studies have indicated that the impact of alcohol on T2D development varies across different beverage types.^26^^,^^27^ Fourth, we did not differentiate between T2D and type 1 diabetes, although it was presumed that nearly all incident diabetes cases in our cohort, which had an age of onset of ≥20 years, were T2D. Fifth, despite accounting for a range of key covariates, the potential for unmeasured confounding factors, such as socioeconomic status,^28^^,^^29^ diet,^30^ and genetic predisposition related to alcohol metabolism (eg, aldehyde dehydrogenase 2 family member, ALDH2),^31^^,^^32^ and T2D^33^ cannot be excluded. Finally, because our study predominantly involved male workers from a single Japanese company, the generalizability of our findings to diverse populations may be constrained. For example, individuals with extremely high levels of alcohol consumption, who might already struggle with alcohol-related substance use disorders, were not likely to be included in our study as such individuals may struggle to maintain employment. This exclusion led to a narrow range of alcohol exposure among our participants, which may have influenced the observed pattern of the association between alcohol consumption and T2D risk.

### Conclusions

While we observed a U-shaped association between alcohol consumption and T2D among individuals with a higher BMI, with the lowest risk observed in those with moderate alcohol consumption, the risk increased linearly with increasing alcohol consumption among individuals with a lower BMI. Further research using recent cohort data from various study populations is required to confirm the observed association, particularly in settings where the prevalence of underweight individuals is not negligible.

## SUPPLEMENTARY MATERIAL

eTable 1. Baseline characteristics of participants according to average daily alcohol consumption category (*n* = 31,524)
