# Supplementary material for: Association Between Alcohol Consumption and the Risk of Type 2 Diabetes Mellitus Across Different Body Mass Index Categories Among Japanese Workers
Source: J Epidemiol. 2025 Aug 5;35(8):364–72. doi: 10.2188/jea.JE20240259 (PMC12237586; doi:10.2188/jea.JE20240259)
Supplement: Supplementary file 1 [file je-35-364-s001.pdf]

eTable 1. Baseline characteristics of participants according to average daily alcohol consumption category (n=31,524)

Characteristics	Whole participants	Alcohol consumption (*go*/day)[a]				
		0	0.1–0.5	0.6–1.0	1.1–2.0	>2.0
N	31,524	10,531	8,340	6,106	5,423	1,124
Age, years, mean [SD]	43.8 [9.3]	42.8 [9.6]	42.0 [9.1]	44.9 [8.8]	46.7 [8.4]	46.6 [8.1]
Sex (men), n (%)	26,819 (85.1)	7,476 (71.0)	7,162 (85.9)	5,782 (94.7)	5,296 (97.7)	1,103 (98.1)
Married, n (%)	23,460 (74.4)	7,004 (66.5)	6,147 (73.7)	4,977 (81.5)	4,475 (82.5)	857 (76.3)
High job position, n (%)	6,161 (19.5)	1,069 (10.2)	1,728 (20.7)	1,567 (25.7)	1,483 (27.4)	314 (27.9)
Smoking status, n (%)						
Never	13,065 (41.4)	5,651 (53.7)	3,936 (47.2)	2,017 (33.0)	1,220 (23.1)	211 (18.8)
Former	5,678 (18.0)	1,185 (11.3)	1,479 (17.7)	1,387 (22.7)	1,366 (25.2)	261 (23.2)
Current	12,781 (40.5)	3,695 (35.1)	2,925 (35.1)	2,702 (44.3)	2,807 (51.8)	652 (58.0)
Occupational physical activity, n (%)						
Mostly sedentary	18,819 (59.7)	5,577 (53.0)	5,478 (65.7)	3,859 (63.2)	3,244 (59.8)	661 (58.8)
Mostly standing	4,578 (14.5)	1,986 (18.9)	994 (11.9)	750 (12.3)	688 (12.7)	160 (14.2)
Mostly walking	5,625 (17.8)	1,948 (18.5)	1,315 (15.8)	1,101 (18.0)	1,057 (19.5)	204 (18.2)
Mostly physically active	2,502 (7.9)	1,020 (9.7)	553 (6.6)	396 (6.5)	434 (8.0)	99 (8.8)
Leisure-time physical activity, min/week, n (%)						
0	20,307 (64.4)	7,411 (70.4)	5,152 (61.8)	3,745 (61.3)	3,258 (60.1)	741 (65.9)
1–59	2,829 (9.0)	831 (7.9)	906 (10.9)	558 (9.1)	459 (8.5)	75 (6.7)
60–119	3,023 (9.6)	816 (7.8)	919 (11.0)	642 (10.5)	550 (10.1)	96 (8.5)
≥120	5,365 (17.0)	1,473 (14.0)	1,363 (16.3)	1,161 (19.0)	1,156 (21.3)	212 (18.9)
Family history of diabetes, n (%)	4,552 (14.4)	1,532 (14.6)	1,193 (14.3)	876 (14.4)	771 (14.2)	180 (16.0)
Body mass index, kg/m^2, mean [SD]	23.3 [3.3]	23.1 [3.6]	23.3 [3.3]	23.3 [3.0]	23.4 [2.9]	23.4 [3.0]
Hypertension, n (%)	4,562 (14.5)	1,163 (11.0)	932 (11.2)	950 (15.6)	1,242 (22.9)	275 (24.5)
Dyslipidemia, n (%)	13,791 (43.8)	4,700 (44.6)	3,562 (42.7)	2,601 (42.6)	2,426 (44.7)	502 (44.7)

SD, standard deviation.

[a] One *go* of Japanese sake contains approximately 23 g of ethanol. The corresponding ranges for alcohol consumption categories, from lowest to highest, were 0; 0.1–11.5 g; 11.6–23.0 g; and ≥23.1 g.
